# Clinical Features, Treatment, and Outcome in 102 Adult and Pediatric Patients with Localized High-Grade Synovial Sarcoma

**DOI:** 10.1155/2011/231789

**Published:** 2011-04-14

**Authors:** H. Al-Hussaini, D. Hogg, M. E. Blackstein, B. O'Sullivan, C. N. Catton, P. W. Chung, A. M. Griffin, D. Hodgson, S. Hopyan, R. Kandel, P. C. Ferguson, J. S. Wunder, A. A. Gupta

**Affiliations:** ^1^Division of Medical Oncology, Princess Margaret Hospital, University of Toronto, Toronto, ON, Canada M5G 2M9; ^2^Division of Medical Oncology, Mount Sinai Hospital, University of Toronto, Toronto, ON, Canada M5G 1Z5; ^3^Department of Radiation Oncology, Princess Margaret Hospital, University of Toronto, Toronto, ON, Canada M5G 2M9; ^4^University Musculoskeletal Oncology Unit, Division of Orthopaedic Surgery, Department of Surgery, Mount Sinai Hospital, University of Toronto, Toronto, ON, Canada M5G 1Z5; ^5^Department of Orthopedic Surgery, Hospital for Sick Children, University of Toronto, Toronto, ON, Canada M5G 1X8; ^6^Department of Pathology, Mount Sinai Hospital, Toronto, ON, Canada M5G 1Z5; ^7^Division of Hematology/Oncology, Hospital for Sick Children, University of Toronto, 555 University Avenue, Toronto, ON, Canada M5G 1X8

## Abstract

*Background*. There remains controversy on the routine use of chemotherapy in localized SS. *Methods*. The records of 87 adult (AP) and 15 pediatric (PP) patients with localized SS diagnosed between 1986 and 2007 at 2 centres in Toronto were reviewed. *Results*. Median age for AP and PP was 37.6 (range 15–76) and 14 (range 0.4–18) years, respectively. 65 (64%) patients had large tumours (>5 cm). All patients underwent en bloc surgical resection resulting in 94 (92.2%) negative and 8 (7.8%) microscopically positive surgical margins. 72 (82.8%) AP and 8 (53%) PP received radiotherapy. Chemotherapy was administered to 12 (13.8%) AP and 13 (87%) PP. 10 AP and 5 PP were evaluable for response to neoadjuvant chemotherapy, with response rate of 10% and 40%, respectively. 5-year EFS and OS was 69.3 ± 4.8% and 80.3 ± 4.3%, respectively, and was similar for AP and PP, In patients with tumors >5 cm, in whom chemotherapy might be considered most appropriate, relapse occurred in 9/19 (47%) with chemotherapy, compared to 17/46 (37%) In those without. *Conclusions*. Patients with localized SS have a good chance of cure with surgery and RT. Evidence for a well-defined role of chemotherapy to improve survival In localized SS remains elusive.

## 1. Introduction

Synovial sarcoma (SS) accounts for approximately 8% of all soft tissue sarcoma (STS) and is more common in adolescents and young adults compared to older individuals [1]. Prognostic factors associated with survival include tumor size [2–7], tumor invasiveness [3–5], stage [5, 7], tumor location [8, 9], histological subtype and grade [3, 7, 9, 10], and incomplete resection as manifested by the pathological resection margin status [11]. Of these, tumour size (greater or less than 5 cm) is the most consistently significant prognostic factor in patients with localized disease [2–7]. Patient age has also been identified as a prognostic factor [7, 9]. Similar to other STS local management for adult patients with localized SS is complete tumour resection, often in combination with either adjuvant or neo-adjuvant radiotherapy. With this approach, the 5-year overall survival approaches 80% in some series [5]. Evidence of a well-defined role for chemotherapy remains uncertain in localized adult STS, but is more debatable in SS. Some series of SS support a survival benefit with chemotherapy [12–14], while others have reached the opposite conclusion [15–18]. Pediatric experience with chemotherapy in SS demonstrates response rates ranging from 37–56%, potentially justifying its use [5, 19]. In the current study, we investigated the impact of chemotherapy on survival in both pediatric and adult patients with localized SS treated at two specialized sarcoma centers.

## 2. Materials and Methods

Between 1986 and 2007, a total of 102 consecutive patients (87 adult and 15 pediatric) with localized SS were treated at the joint Mount Sinai Hospital/Princess Margaret Hospital Sarcoma Program (adult patients) and The Hospital for Sick Children (pediatric patients), Toronto, ON, Canada. Patients included in this study received all definitive sarcoma therapy (surgery, chemotherapy, and/or radiation therapy) at the two respective institutions. Patients were included in the study if they presented with a localized primary malignancy with no evidence of lung metastases and had not previously received any tumour therapy. After institutional review board approval, medical records at each center were reviewed and data on age at diagnosis, tumor-specific data (histology, size (<5 cm or ≥5 cm), location, depth, grade, surgical margins, and lymph node status), therapy (chemotherapy, radiation, and surgery), and clinical outcome were collected. Both pediatric and adult patients had cross-sectional imaging of their primary tumour (most commonly MRI) as well as chest imaging (most commonly CT scan of the chest). A tumor was considered as invading bone or neurovascular structures if there was either gross or microscopic invasion at pathologic examination. Information on specific histological subtype was not available for many cases and thus is not included in this paper. Response to chemotherapy was assessed using RECIST criteria [20] in those patients who had both pre- and postchemotherapy MRI or CT scans performed prior to surgery and preoperative radiotherapy, if it was utilized. The total number of cycles of chemotherapy administered prior to re-evaluation varied between patients.

All adult and pediatric patients underwent definitive surgical resection. The delivery of radiotherapy and chemotherapy varied between the adult and pediatric hospitals, but was determined at a multidisciplinary sarcoma tumor board conference. During the course of this study, if an adult patient was treated with preoperative radiotherapy, 50 Gy in 2 Gy daily fractions was administered. Until the year 2000, there was an additional possibility for a 16 Gy in 2 Gy per fraction postoperative boost if the surgical resection margins were positive. Patients treated with postoperative radiotherapy received 66 Gy. Radiation was generally utilized when wide surgical resection margins were not attainable [21]. In children, radiotherapy was reserved for those cases with microscopic positive margins. In adults, the majority of chemotherapy included both doxorubicin and ifosfamide, whereas the protocol was more varied in children. 

### 2.1. Statistical Methods

Survival rates were determined using the Kaplan and Meier technique [22]. Event-free survival (EFS) was defined as time between diagnosis and relapse or death from any cause. Overall survival (OS) was defined as time between diagnosis and death due to any cause. Survival curves were compared between different groups using the log-rank test. Fisher's Exact test was used to compare categorical variables in univariate analysis using SPSS v 17.0 (SPSS Inc, Chicago, IL).

## 3. Results

There were 87 adult and 15 pediatric patients (*n* = 102) with a median follow-up time of 5.6 years (range 0.26–18 years). The median age for adult and pediatric patients was 37.6 (range 15 to 76) and 14 (range 0.4 to 18) years, respectively. There were 5 patients less than age 18 (15, 16, 17 years) who were treated at the adult center and are therefore included in the adult cohort. The most common site for the primary tumor for all patients was the lower extremity (*n* = 58, 57%). Sixty-five (64%) patients had large tumors (≥5 cm), 10 (9.8%) had bone invasion, and 6 (5.9%) had evidence of neurovascular invasion. (Table 1) All tumours were high grade. All patients underwent en bloc surgical resection resulting in 94 (92.2%) negative and 8 (7.8%) microscopically positive surgical margins. Twelve (13.8%) of adult patients had primary amputation—8 below knee, 2 forequarters, 1 below elbow, and 1 above knee. Seventy-two (82.8%) adult and 8 (53%) pediatric patients received radiotherapy. The median radiation doses were 50.4 Gy (range 50 to 66) for adult and pediatric patients. 

Chemotherapy was administered to 25 (24.5%) patients, 12 (13.8%) adult and 13 (87%) pediatric. The median number of chemotherapy cycles delivered was 5 and 7, for adult, and pediatric patients, respectively. The most common chemotherapeutic regimen administered was doxorubicin-based in 22 patients. The median total dose of doxorubicin was 300 mg/m^2^ (range 150 to 375) and 265 mg/m^2^(range 90 to 375) for adult and pediatric patients, respectively (target dose per cycle 75 mg/m^2^). The median total dose of ifosfamide was 25050 mg/m^2^ and 23260 mg/m^2^ for adult (*n* = 9) and pediatric (*n* = 13) patients, respectively (target dose per cycle 5 g/m^2^). (Table 2) Nine (75%) adults and 10 (77%) pediatric patients received both anthracycline and an alkylating agent. Among 12 adult patients who received neo-adjuvant chemotherapy, response was evaluated in 10. After a median of 3 cycles of preoperative chemotherapy, there were 8 patients with stable disease, 1 partial response, and 1 case with progressive disease, for a response rate of 10%. Five of thirteen pediatric patients had repeat imaging after a median of 2 cycles of chemotherapy. Response evaluated included 2 cases of stable disease, 1 partial response PR, 1 progressive disease, and 1 complete response, for a response rate of 40%. 

The estimated 5-year event-free survival (EFS) and overall survival (OS) for the entire group were 69.3 ± 4.8% and 80.3 ± 4.3%, respectively. The 5-year EFS for adult and pediatric patients was 68.3 ± 5.2% and 74.9 ± 13% (*P* = .33), respectively. The 5-year OS for adult and pediatric patients was 76.9 ± 5.0% and 100 ± 27.2% (*P* = .36), respectively. Disease relapse occurred in 32 (31.4%) patients (29/87 adults and 3/15 children): 28 (27.4%) had a distant recurrence in the lung, 3 (2.9%) had a local recurrence, and 1 (1.0%) developed concurrent local and distant relapse. The overall rate of local disease recurrence was 4/102 (3.9%). 

**Figure 1:**
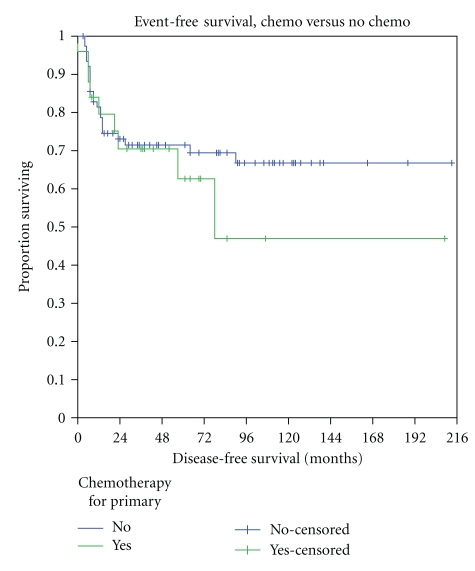
Event-free survival of all patients who did and did not receive chemotherapy. blue line—no chemotherapy; Green line—with chemotherapy.

Patients with large tumors had significantly worse EFS (61.5 ± 6.4%) compared to patients with smaller lesions (81.9 ± 6.7%, *P* = .03), and this was almost entirely related to distant metastasis. (Table 3) The presence of bone invasion was also associated with worse EFS (45 ± 17.4% versus 74.5 ± 5.1%, *P* = .02). The presence of neurovascular invasion was not associated with worse EFS (60 ± 21.9% versus 71.5 ± 5.2%, *P* = .84). The effect of chemotherapy was initially assessed in the entire cohort. Of the patients who received chemotherapy, 9/25 (36%) relapsed (3/13 children and 6/12 adults) compared to 23/77 (30%; all adults) for patients who did not receive chemotherapy. 5-year EFS was similar in patients who received or did not receive chemotherapy (62.6 ± 11.2% versus 71.5 ± 5.3%) (Figure 1). In patients with tumors ≥5 cm in whom chemotherapy might be considered most appropriate, relapse occurred in 9/19 (47%) of those who received chemotherapy compared to 17/46 (37%) in those who did not receive chemotherapy. Event-free survival was not significantly different between these 2 groups (*P* = .37). Margin status, depth, radiation therapy did not influence EFS. There was no difference in EFS between patients treated at the adult versus pediatric center, or between those greater or less than age 18 or between those greater or less than age 30, irrespective of treating hospital.

## 4. Discussion

In the setting of an adult and pediatric tertiary care center, each with expertise in sarcoma management, the overall and event-free survival rates for 102 patients with localized SS were 80% and 69%, respectively, with no difference between pediatric and adult patients, nor between those who did or did not receive chemotherapy. Our data failed to demonstrate that pediatric patients with localized SS have a better outcome than adults or that routine use of chemotherapy is beneficial in reducing systemic relapse, even in patients with large tumours. Although our findings are limited by the nonrandomized delivery of chemotherapy and the small sample size, this study contributes to the growing literature questioning the routine use of chemotherapy in localized SS [15, 16, 18, 23, 24]. 

Much of the current support for using chemotherapy as part of the curative treatment protocol for management of patients with localized but high-risk soft tissue sarcoma arose following a randomized study by Frustaci et al. [25]. Unfortunately, the early promising results showing an improvement in overall survival following treatment with chemotherapy did not hold up with longer followup [26]. A recent meta-analysis of chemotherapy in STS did identify a marginal benefit of doxorubicin and ifosfamide treatment [27], although histologic subtype analyses were not performed. However, other studies specific to SS have continued to support a role for chemotherapy. Widemann et al. reported 5-year metastasis-free survival rates of 60% compared to 48% for those patients with localized SS who did or did not receive chemotherapy, respectively, although no statistical analysis was provided [28]. However, there was no difference in overall survival rates between the two treatment arms in that study. Eilber et al. reported a 4-year distant relapse-free survival rate of 74% versus 46% (*P* = .01), and disease-specific survival of 88% compared to 67% in patients with SS treated with or without ifosfamide-based chemotherapy, respectively [14]. However, it is interesting to note that in our study, the 5-year overall survival of adults who did not receive chemotherapy (75.6%) was comparable to the chemotherapy treatment arms in these two studies. Patients with SS are reported to have late relapses (after 6 years) [13], an event which would not have been uniformly captured in our series.

In comparison to the above results, other studies have refuted the role of systemic therapy for SS. A study of 237 patients from the French Sarcoma Group found that chemotherapy had no significant impact on outcome [15]. An analysis of 250 patients with SS treated at the Rizzoli Institute also failed to show any improvement in survival with chemotherapy, even using high doses of alkylating agents (e.g., ifosfamide 9 g/m^2^/cycle) [17, 23]. In our study, adult patients received a median total cumulative dose of ifosfamide of 25 g/m^2^or just over 5 g/m^2^ per cycle for 5 cycles. Although there are reports of patients with metastatic SS responding to even higher doses of ifosfamide (e.g., 14 g/m^2^/cycle) [29], this strategy has not been shown to improve survival [30, 31]. 

Compared to other STS, SS has been considered a “more chemosensitive” subtype as previous studies have documented favorable response rates to chemotherapy [28, 32]. In our study, the response rate to neo-adjuvant chemotherapy was disappointing, particularly in adults. In comparison, the 40% response rate to chemotherapy observed in the pediatric patients in our series was comparable to previously published reports of 37% [5] and 56% [19], but did not translate into a survival advantage, similar to our adult group. Our evaluation of response to therapy was imperfect due to the differing number of cycles administered to each patient prior to radiological re-evaluation. Furthermore, there may be limitations in using RECIST to evaluate response to therapy in STS [33–36].

The 5-year EFS of 75% for children in our series is comparable to rates of 74.3% [12] and 72% [5] reported by others. The chemotherapy regimens delivered in these pediatric series were variable, although the total cumulative doses of ifosfamide and doxorubicin were similar to the adult and pediatric patients of our study. Although the majority of pediatric protocols currently offer chemotherapy for SS, there is a lack of proven benefit for this approach [4, 5]. For example, in one large series, the 5-year EFS for pediatric patients with localized SS, all of whom received chemotherapy, was 41%, compared to 61% for all patients (adult + children) in our study [6].

We report a very low local failure rate of 3.9% (4/102) compared to other large studies which reported local recurrence in 18% [23], 23.5% [15], and 28% [28] of patients with localized and resectable SS. This is likely due to the combination of a high rate of negative surgical margins (92%), as well as the fact that the majority of adults and approximately half the pediatric patients in our study were treated with radiotherapy in a specialized sarcoma setting by only a small number of radiation oncologists, which seems to have a bearing on local control rates [37, 38]. Although neither margin nor RT status were associated with the risk of systemic relapse in our study, this may have been due to the small number of cases which did not receive radiation or had positive resection margins. However, these two factors are strongly correlated with the risk of local relapse [15, 23, 28]. Although the impact of local recurrence on development of metastasis is controversial for STS, there is certainly support for a causative effect [39–42]. The low local recurrence rate in this study may partially explain our favourable survival rates. 

We identified tumour size as the most important predictor of systemic outcome, similar to almost every other investigation of prognostic factors for SS. Patients with bone invasion were also found to have worse outcomes, similar to the findings by both Ferguson et al., [43] and Panicek et al., who showed that bone invasion, identified either pathologically or by MR imaging, respectively, was associated with worse overall survival in STS [44]. In comparison, Panicek et al. and Ghert et al. showed that vascular invasion or encasement was not a significant predictor of outcome in soft tissue sarcoma [44, 45]. 

The results of this study show that a well-planned local therapy regimen including wide surgical resection, with or without radiotherapy as necessary, is effective in preventing local relapse of SS. Unfortunately, the addition of chemotherapy did not lead to an improvement in the rates of systemic recurrence. Our patients with localized SS had a very good chance of curative treatment with surgery and radiation alone, even if their tumours were large. Since this was true for both adult and pediatric patients [4, 46], it suggests that the treatment approach for the different age groups should converge, recognizing that in some instances, particularly for pediatric patients, there may be a higher risk of morbidity due to the potentially detrimental effects of radiation on skeletal growth. The short- and long-term toxicities of chemotherapy must be weighed against the morbidities associated with radical surgery, with or without radiotherapy. The long-term effects of alkylating agents are most important in the younger pediatric cohort, in whom fertility preservation is a challenge for prepubertal boys [47, 48] and in whom the magnitude of anthracycline cardiotoxicity is well documented [49]. Chemotherapy should not be automatically offered to adult or pediatric patients with localized SS. Rather, investigators should continue to strive to develop novel agents that may directly target the pathways affected by the SYT-SSX translocation and develop more effective techniques of delivering systemic therapy.

## Figures and Tables

**Table 1:** Patients and tumor characteristics.

Characteristic	Adult center (%)	Pediatric center (%)	Total (%)	*P*
Age				.0003
≤5 years	0 (0)	2 (13.3)	2 (2.0)	
6–17 years	5 (5.7)	12 (80)	17 (16.7)	
18–40 years	44 (50.6)	1 (6.7)	45 (44.1)	
>40 years	38 (43.7)	0 (0)	38 (37.2)	

Gender				.16
Female	42 (48.3)	4 (26.7)	46 (45)	
Male	45 (51.7)	11 (73.3)	56 (55)	

Tumour size				.5
<5 cm	31 (35.6)	4 (26.7)	35 (34.3)	
≥5 cm	56 (64.4)	9 (60)	65 (63.7)	
unknown	0	2 (13.3)	2 (2.0)	

Depth				.69
Superficial	11 (12.6)	1 (6.7)	12 (11.8)	
Deep	75 (86.2)	14 93.3)	89 (87.2)	
Unknown	1 (1.1)	0 (0)	1 (1%)	

Site				.0001
Upper extremity	26 (29.9)	2 (13.3)	28 (27.4)	
Lower extremity	53 (60.1)	5 (33.3)	58 (56.9)	
Pelvic	0 (0)	1 (6.7)	1 (1)	
Shoulder	0 (0)	1 (6.7)	1 (1)	
Other	8 (92)	6 (40)	14 (13.7)	

Margins				.85
Negative	80 (92.0)	14 (93.3)	94 (92.2)	
Microscopic positive	7 (8.0)	1 (6.7)	8 (7.8)	

Bone invasion				.2
Present	10 (11.5)	0 (0)	10 (9.8)	
Absent	65 (74.7)	15 (100)	80 (78.4)	
Unknown	12 (13.8)	0 (0)	12 (11.8)	

Neurovascular Invasion				.58
Present	6 (6.9)	0 (0)	6 (5.9)	
Absent	69 (79.3)	15 (100)	84 (82.3)	
Unknown	12 (13.8)	0 (0)	12 (11.8)	

Radiation therapy				.01
Yes	72 (82.8)	8 (53.3)	80 (78.4)	
No	14 (16.1)	7 (46.7)	21 (20.6)	
Unknown	1 (1.1)	0 (0)	1(1)	

Chemotherapy				.0004
Yes	12 (13.8)	13 (86.7)	25 (24.5)	
No	75 (86.2)	2 (13.3)	77 (75.5)	

**Table 2:** Total cumulative dose of chemotherapy received.

	Adult center	Pediatric center		
	Median (range)		Median (range)	
	mg/m^2^	*n*	mg/m^2^	*n*
Chemotherapy				
Doxorubicin	300 (150–375)	12	265 (90 – 375)	10
Ifosfamide	25050 (15030–39800)	9	23260 (7500–65600)	13
Cyclophosphamide	14590 (5180–24000)	2	4980 (1200–15260)	10

**Table 3:** Univariate analysis of prognostic factors in adult and pediatric SS.

Variable		5-year EFS	*n*	*P*
Size	<5 cm	62 ± 6.3%	35	.03
	≥5 cm	82 ± 6.7%	65	

Depth	Superficial	81.8 ± 11.6%	12	.35
	Deep	69.1 ± 5.1%	89	

Microscopic margin status	Positive	62.5 ± 17.1%	94	.62
	Negative	70.4 ± 5.0%	8	

Center	Pediatric	74.9 ± 13.0%	15	.31
	Adult	68.7 ± 5.1%	87	

Age	≤30	70.9 ± 6.7%	51	.47
	>30	68.6 ± 6.8%	51	

Bone invasion	Present	45 ± 17.4%	10	.02
	Absent	74.5 ± 5.1%	80	

Neurovascular invasion	Present	60.0 ± 21.9%	6	.87
	Absent	71.8 ± 5.2%	84	

Radiation	Yes	69.6 ± 5.4%	80	.67
	No	67.5 ± 11%	21	

Chemotherapy	Yes	62.6 ± 11.2%	25	.48
	No	71.5 ± 5.3%	77	

Adults	Chemotherapy	56.3 ± 14.8%	12	.13
	No chemo	70.7 ± 5.4%	75	

Pediatric	Chemotherapy	69.2 ± 15.6%	13	.41
	No chemo	100%	2	

>5 cm	Chemotherapy	51.3 ± 13.4%	19	.37
	No chemo	65.7 ± 7.2%	46	
